# Deregulation of the CEACAM Expression Pattern Causes Undifferentiated Cell Growth in Human Lung Adenocarcinoma Cells

**DOI:** 10.1371/journal.pone.0008747

**Published:** 2010-01-18

**Authors:** Bernhard B. Singer, Inka Scheffrahn, Robert Kammerer, Norbert Suttorp, Suleyman Ergun, Hortense Slevogt

**Affiliations:** 1 Institute of Anatomy, University Hospital Essen, Essen, Germany; 2 Institute of Immunology, Friedrich Loeffler Institute, Tuebingen, Germany; 3 Department of Internal Medicine/Infectious Diseases and Pulmonary Medicine, Charité-Universitätsmedizin Berlin, Berlin, Germany; School of Medicine, University of Barcelona, Spain

## Abstract

CEACAM1, CEA/CEACAM5, and CEACAM6 are cell adhesion molecules (CAMs) of the carcinoembryonic antigen (CEA) family that have been shown to be deregulated in lung cancer and in up to 50% of all human cancers. However, little is known about the functional impact of these molecules on undifferentiated cell growth and tumor progression. Here we demonstrate that cell surface expression of CEACAM1 on confluent A549 human lung adenocarcinoma cells plays a critical role in differentiated, contact-inhibited cell growth. Interestingly, CEACAM1-L, but not CEACAM1-S, negatively regulates proliferation via its ITIM domain, while in proliferating cells no CEACAM expression is detectable. Furthermore, we show for the first time that CEACAM6 acts as an inducer of cellular proliferation in A549 cells, likely by interfering with the contact-inhibiting signal triggered by CEACAM1-4L, leading to undifferentiated anchorage-independent cell growth. We also found that A549 cells expressed significant amounts of non-membrane anchored variants of CEACAM5 and CEACAM6, representing a putative source for the increased CEACAM5/6 serum levels frequently found in lung cancer patients. Taken together, our data suggest that post-confluent contact inhibition is established and maintained by CEACAM1-4L, but disturbances of CEACAM1 signalling by CEACAM1-4S and other CEACAMs lead to undifferentiated cell growth and malignant transformation.

## Introduction

Lung cancer is the most common cause of cancer and cancer related mortality worldwide[1]. Tumor-progression is characterized by the invasion of tumor cells into the surrounding tissue. Their ability to form metastases in distant organs is a poor prognostic factor indicating disease dissemination[2]. The vast majority of primary lung tumors are carcinomas, derived from epithelial cells. Although their genetic and phenotypic properties are still not well defined[3]–[5], various cell adhesion molecules (CAMs) appear to play a critical role in the processes of invasion and metastasis[6], [7]. Normally CAMs tightly orchestrate tissue structure by their ability to mediate cell-cell interaction and contact inhibition. Contact inhibition describes the process of arresting proliferation when cells come into contact with each other. Thus, the cells remain in a confluent layer with no piling up or dissociation of cells from the monolayer. In the case of malignant transformation, alterations of CAM expression and function occur. Members of the carcinoembryonic antigen (CEA) related cell adhesion molecule (CEACAM) family are known to be deregulated in various tumors, and represent one example of such proteins. CEACAMs are expressed in normal epithelia, angiogenically activated endothelia, and haematopoetic cells, and mediate homotypic and heterotypic cell-cell interactions[8]–[10]. The functions mediated by CEACAMs differ widely depending on the growth stage and activation status of the cell. A number of CEACAM family members have been found to be involved in the processes of cancerous growth and invasion defining them as either tumor suppressors or as poor prognostic markers for the progression of malignancies[10]–[13]. CEACAMs belong to the immunoglobulin (Ig) superfamily and thus appear as highly glycosylated proteins with the typical N-terminal variable Ig-like domain followed by 0 to 6 constant Ig-like domains, and either a hydrophobic transmembrane domain with a cytoplasmic tail (CEACAM1-CEACAM4), or a glycosylphosphatidylinositol (GPI) lipid moiety (CEACAM5-CEACAM8)[14]–[16]. In epithelia, four distinct CEACAMs have been demonstrated, namely CEACAM1, CEACAM5, CEACAM6 and CEACAM7[15]. Furthermore, CEACAM1 occurs in four major splice variants with either three or four Ig domains and either a short (S) or a long (L) cytoplasmic domain consisting of 12 aa or 73 aa residues, respectively, giving the following variants: CEACAM1-3S, CEACAM1-4S, CEACAM1-3L and CEACAM1-4L[15], [17]. The cytoplasmic domain of CEACAM1-3/4L possesses an immunoreceptor tyrosine-based inhibition motif (ITIM) which contains two tyrosine residues that can recruit and activate SH2 domain-containing tyrosine kinases and tyrosine phosphatases[18], [19]. The interactions of CEACAM1-L with these enzymes play an important role for regulating signaling cascades and cellar functions [18], [20]. Although CEACAM1-S lacks tyrosine residues in its cytoplasmic domain, it also mediates cell-cell interactions[9], [21].

Different members of the CEACAM family or the different isoforms of CEACAM1 can have different activities[8], [14]. However, little is known about the differential expression patterns of CEACAMs and their role for cell-cell interactions in pulmonary epithelial cells. Interestingly, Laack et al. identified CEACAM1 expression as a novel prognostic marker in adenocarcinomas of the lung. In this study patients with adenocarcinomas expressing CEACAM1 had a significantly worse overall and disease-free survival in comparison with those who had a CEACAM1-negative tumor[22]. In addition, Sienel et al. reported that elevated CEACAM-1 expression in primary non small cell lung cancer (NSCLC) correlates with lung cancer progression[13]. The human lung adenocarcinoma cell line A549 represents a well established model for pulmonary epithelial cells[20], [23]–[25]. Although these cells are known to expresses CEACAM1[20], [26], [27], the exact expression pattern and function of CEACAMs in this cell line are not yet defined. Like other carcinoma cell lines, A549 cells are a non-homogenous cell population with several subpopulations, each with various characteristics[3], [28], [29]. Interestingly, Croce et al. reported specific findings about morphological and functional heterogeneity in four different A549 subpopulations[3]. They demonstrated that three A549 subpopulations, which represented the non-tumorigenic (NT) A549 cells with retained characteristics of more differentiated adenocarcinomas, formed well spread monolayers, exhibited decent contact inhibition, and were not tumorigenic in a nude mice system. In contrast, cells of a fourth, more undifferentiated subpopulation, also demonstrated normal cobblestone-like epithelial growth during proliferation to a confluent monolayer, however, some cells overcame contact inhibition and exhibited anchorage independent growth on top of the A549 monolayer. Importantly, cells of this population were demonstrated to be tumorigenic in nude mice and thus were named tumorigenic (T) A549 cells[3].

These observations prompted us to investigate the relationships of the expression pattern and function of different CEACAMs with proliferation and contact mediated growth inhibition in A549 cells. In addition, the role of the CEACAM1-L and -S splice variants and the cytoplasmic ITIM domain in cellular morphology and growth behavior were studied. Our results suggest that the expression pattern and the interplay of CEACAM1 with different CEACAMs are of central importance for the regulation of proliferation and contact inhibition. Thus, CEACAM mediated functions appear to be important regulators of the invasive and metastatic behavior of tumor cells.

## Results

### The Non-Tumorigenic A549-NT and the Tumorigenic A549-T Subpopulations Differed in Contact-Mediated Growth Inhibition Properties

First we isolated distinct A549 subpopulations by density gradient centrifugation as described by Croce et al.[3] and characterized their CEACAM expression pattern. Density gradient centrifugation of A549 cells resulted in four different subpopulations: subpopulation A at the top of the gradient in fraction 0–3 corresponding to the density of 1.035, subpopulation B in the fraction 4–6 (density 1.040), subpopulation C in fraction 7–9 (density 1.050) and subpopulation D in fraction 10–12 (density 1.060). Cells accumulate at higher density fractions due to their size and granularity, which usually reflects an activated cell status. In accordance with published results, we could subdivide A549 cells in two groups as evidenced by their morphology and growth behavior. One corresponded to the non-tumorigenic like subpopulations A549-A, -B and -C, and the other to the tumorigenic A549-D[3]. To simplify our further studies we combined the subpopulations A549-A, -B and -C and named them A549-NT (non-tumorigenic) and A549-T (tumorigenic), respectively.

**Figure 1:**
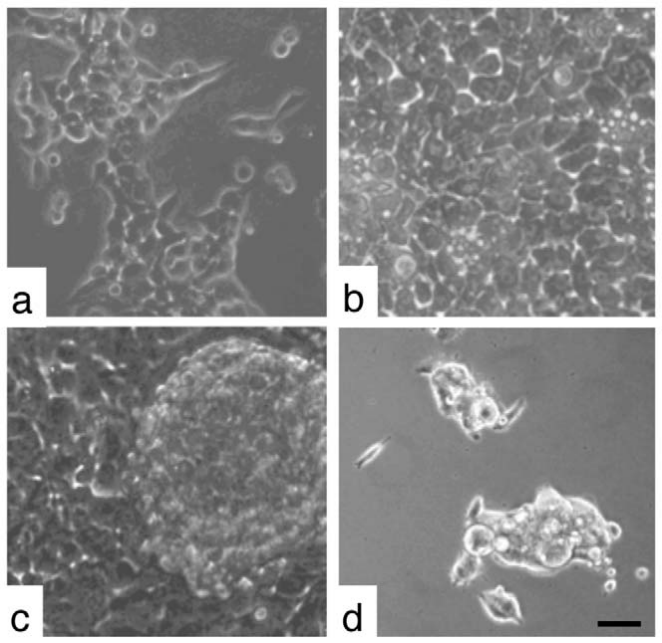
Representative images of different A549 cell clones demonstrating their morphological heterogeneity by phase contrast imaging. Subpopulation A549-NT grown exponentially (a), to tight confluence forming cell-contact inhibited monolayers (b). Tight confluent subpopulation of A549-T (c) piled up on the monolayer revealing the ability to survive and proliferate independently of cell-matrix anchorage, leading to detached spheroidal colonies on top of the monolayer. (d) Spheroidal A549-T colonies seeded into fresh culture vessels attached and grew as monolayers. Bar, 50 µm.

Phase-contrast microscopy revealed that under standard culture conditions sub-confluent A549-NT and -T occurred as scattered colonies of associated cells (Figure 1A). However, confluent A549-NT cells appeared as cobblestone monolayer, characteristic of epithelial cell growth, consistent of closely associated cells with a typical polygonal, epithelial-like morphology indicating a stringent contact inhibition of cell growth (Figure 1B). In addition, this subpopulation formed tight cell-cell contacts leading to a significant prolongation of the trypsin treatment necessary for harvesting if compared with the time needed to harvest exponential growing A549-NT cells. The A549-T subpopulation also grew as a normal epithelial monolayer. However, in contrast to the A549-NT, some cells piled up on each other showing anchorage independence. These cell aggregates were found mainly at the top of the monolayer together with suspended cells (Figure 1C). Over time, the spheroid aggregates detached, floated unbound on top of the monolayer and formed proliferating colonies containing approximately 4–400 cells. After seeding A549-T spheroids into new culture vessels, most of the cells attached within a few hours (Figure 1D), grew to a confluent monolayer and again generated detached spheroids of growing cells (data not shown). A549-NT cells had a doubling time of approximately 24 hours and A549-T cells of approximately 20 hours as determined by cell cycle determinations (data not shown).

### The A549-NT and A549-T Subpopulations Revealed Different CEACAM Expression Patterns

**Figure 2:**
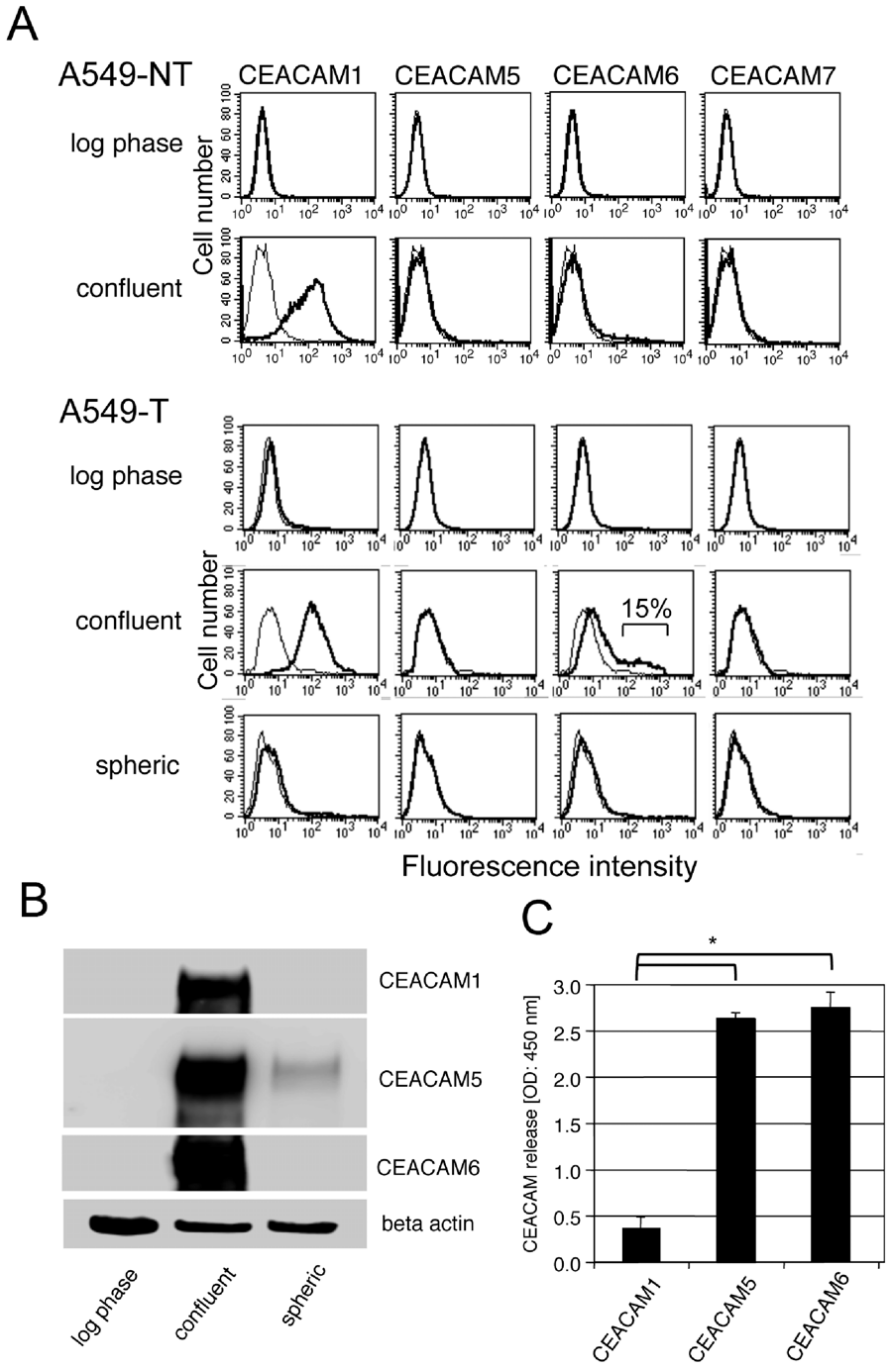
Characterization of the cell surface expression of different CEACAMs in A549-NT and A549-T cells. A) A549 subpopulations collected from different cell growth stages (as indicated) were stained for CEACAM1 with mAb clone 283340, for CEACAM5 with Col-1, for CEACAM6 with mAb 9A6 and for CEACAM7 with BAC2 (thick line). The background fluorescence was determined by incubating the cells with control IgG antibody instead of primary anti-CEACAM antibody (thin line). Subsequently, samples were analyzed by flow cytometry revealing CEACAM1 expression in confluent A549-NT and A549-T cells. Additionally, a minor fraction of confluent A549-T cells expressed CEACAM6. The data shown are representative of three independent experiments. B) Determination of different CEACAMs in whole cell lysates of A549 cells cultured in the non-confluent log phase, the confluent phase and the spheroidal unanchored growing cells by immunoblotting with mAb specific for CEACAM1, CEACAM5 and CEACAM6. The detection of beta-actin served as a loading control. The data shown are representative of three different experiments. C) Soluble CEACAM1, CEACAM5 and CEACAM6 forms are released in the cell culture supernatant of confluent A549 cells. The released CEACAM1, CEACAM5 and CEACAM6 molecules were quantified by specific sandwich ELISA as described in the Materials and Methods section. The mean values ± SD (*p≤0.005) were determined from triplicates. The experiment was repeated twice.

These observations concerning different growth properties of the A549 subpopulations led us to assume that different expression patterns of CEACAMs might be important in the loss of contact inhibition in these cells. Subsequently, the cell surface expression of CEACAM1, CEACAM5, CEACAM6, and CEACAM7 was measured on trypsin-dissociated A549-NT and A549-T cells harvested from non-confluent, confluent monolayers, or - in the case of A549-T cells, – cells growing as spheroids, by flow cytometry. As shown in Figure 2, neither proliferating cells of subpopulations cultivated in the log phase, nor A549-T cells growing as spheroids, expressed CEACAM1, CEACAM5, CEACAM6, or CEACAM7 on their cell surface. In contrast, in the tightly confluent monolayer of both the A549-T and A549-NT cell subpopulations, we found significant cell surface expression of CEACAM1, but not CEACAM5, in all A549 cells (Figure 2A). However, while cells of the A549-NT subpopulation also did not express cell surface bound CEACAM6, around 15% of the A549-T cells expressed significant levels of CEACAM6 on their cell surface (15%±6%, n = 7; Figure 2, lower panel). Western blot analyses confirmed that non-confluent A549 cells did not express CEACAM1, CEACAM5, or CEACAM6 (Figure 2B). Confluent A549 cells showed significant levels of CEACAM1, but in addition revealed a significant expression of CEACAM5 and CEACAM6 as well (Figure 2B). Thus, CEACAM5 and CEACAM6 proteins were expressed in confluent A549 cells, but apparently they did not appear at the cell surface except for some CEACAM6 in a minor fraction of cells (Figure 2A). To elucidate the discrepancy in our findings, we analyzed the cell culture supernatant of confluent A549 cells. We found that both CEACAM5, CEACAM6, and, to a minor extent, CEACAM1, were released into the cell culture supernatant of confluent A549-NT (Figure 2C) and A549-T cells (data not shown).

### CEACAM1 Is Mainly Expressed in the Post-Confluent Growth Phase of A549 Cells

**Figure 3:**
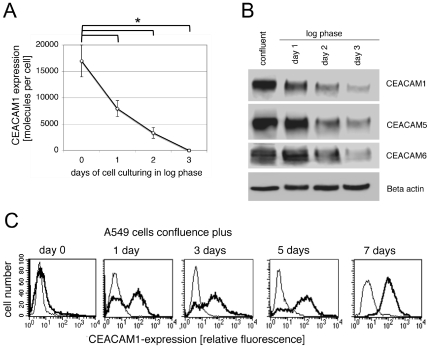
CEACAM1 expression differs in contact-inhibited and proliferating A549 cells. A) For quantification of CEACAM1 cell surface expression in confluent and log phase cultured A549 cells, samples were analyzed by flow cytometry utilizing the QuiFiKit approach as described in Materials and Methods. Briefly, cells were stained for CEACAM1 with mAb clone 283340. Fluorescence was quantitated using QuiFiKit calibration-beads as described in Materials and Methods. The data shown are means ± SD (*p≤0.007) of three different experiments. B) Immunoblot analyses of confluent and proliferating A549 cell lysates were done as described in Materials and Methods using monospecific mAbs directed against CEACAM1 (clone 283340), CEACAM5 (Col-1), and CEACAM6 (9A6), respectively and visualized by HRP-coupled secondary antibody and ECL detection. Beta-actin served as a loading control. The blots shown are representative of three separate experiments. C) The fraction of CEACAM1 expressing A549 cells increased over time as cells were kept in the confluent state. To analyze the CEACAM1 expression in A549 cells grown to confluence (day 0), plus 1 day, plus 3 days, plus 5 days and plus 7 days, cells were stained with mAb clone 283340 (thick line) and isotype matched control antibody (thin line) followed by FITC-conjugated secondary antibody. Subsequently, samples were measured by flow cytometry. The data shown are representative of three different experiments.

To analyze the regulation of the distinct expression patterns of CEACAMs found in altered growth stages in more detail, tightly confluent un-separated A549 cells were seeded into culture vessels with low cell density. Then cells were constantly cultured in exponential growth phase for one, two and three days, respectively. Initially, absolute CEACAM1 expression on the surface of confluent A549 cells was analyzed by quantitative flow cytometry. As shown in Figure 3A an approximate expression of 17,000±3020 (n = 4) molecules per cell was observed. One day later (day 1) the CEACAM1 expression dropped to 6600±1523 (n = 4) molecules per cell, and continued to decrease until no CEACAM1 expression on the cell surface was detectable on day 3 (Figure 3A). Further flow cytometric analyses of cells that were kept non-confluent for up to 10 days, revealed that CEACAM1 did not appear on the cell surface (data not shown). This finding was confirmed by Western blotting, while some minor CEACAM1 expression in lysates of exponential proliferating A549 cells harvested on day 3 was still detectable (Figure 3B). In addition to the significant decrease of CEACAM1 expression, a clear reduction of CEACAM5 and CEACAM6 protein from day zero to day three was found (Figure 3B). Thus, cell-cell contact seemed to play an important role in regulating the expression of CEACAMs in both A549 subpopulations.

Next, we investigated the kinetics of CEACAM1 expression from just confluent A549 cells to up to seven days post-confluence. As shown in Figure 3C, we found that A549 cells that had just reached confluence did not express CEACAM1. However, as soon as A549 cells entered the post-confluent growth arrest state, an increasing fraction of cells started to express high amounts of CEACAM1. At day seven expression of CEACAM1 could be detected on every cell of the A549 monolayer (Figure 3C). According to these results, not only the initial cell-cell contact, but also entering the growth arrest state, precedes the cell surface expression of CEACAM1. Thus CEACAM1 does not cause, but rather stabilizes and maintains contact inhibition. The expression of CEACAM5 and CEACAM6 in the A549 cells also varied with cell confluence in a similar manner (data not shown).

### A549-NT and A549-T Cells Expressed CEACAM1 Isoforms at a Similar Ratio of Total L Compared to Total S Isoforms

**Figure 4:**
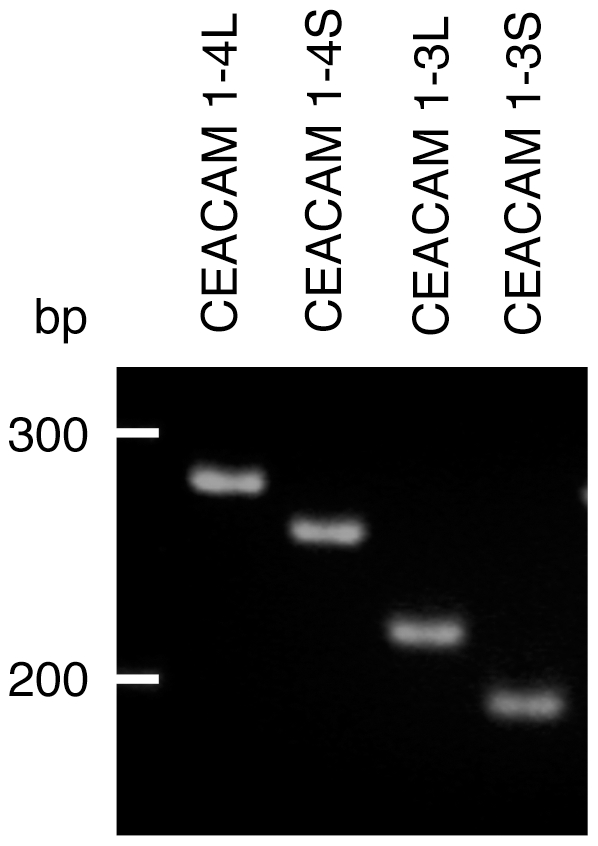
Four CEACAM1 splice variants are expressed in confluent A549 cells. RT of RNA isolated from confluent A549 cells were applied to four different PCR reactions using primer pairs specific four each of the four CEACAM1 isoforms. Products corresponding to CEACAM1-4L (266 bp), CEACAM1-4S (245 bp), CEACAM1-3L (177 bp), and CEACAM1-3S (145 bp), respectively, were amplified. The size of oligonucleotide markers is shown on the left.

In principle four different CEACAM1 splice variants are known to exist in human epithelia, namely CEACAM1-3S, CEACAM1-4S, CEACAM1-3L and CEACAM1-4L. To analyze these splice variants in tightly confluent A549-NT and A549-T cells, we established four different quantitative RT-PCRs, which discriminated between these splice variants. We found that post-confluent contact inhibited A549 cells of both subpopulations expressed all four of these CEACAM1 isoforms (Figure 4 and Table 1). CEACAM1-4L appeared to be the predominant isoform expressed followed by CEACAM1-3S (Table 1). CEACAM1-4S and CEACAM1-3L were expressed at much lower levels. In general, the sum of the expression of the two L isoforms (∑**L**) was greater than the sum of the two S isoforms (/∑**S**), and there was no significant difference between the two subpopulations A549-NT and A549-T with respect to the ∑L/∑S ratio.

**Table 1:** Characterization of the CEACAM1 isoform pattern as determined by quantitative RT-PCR of the two A549 subpopulations.

	A549-NT	A549-T
	Confluent/G0	Confluent/G0
CEACAM1-4L	104.600±47.600	74.970±33.000
CEACAM1-4S	23.490±1.420	9.250±5.570
CEACAM1-3L	13.300±1.980	7.590±2.930
CEACAM1-3S	58.870±3.470	26.330±7.430
	[Copies/µg]	[Copies/µg]

### CEACAM1-4L Induces Contact-Mediated Inhibition of Cell Growth in A549 Cells

**Figure 5:**
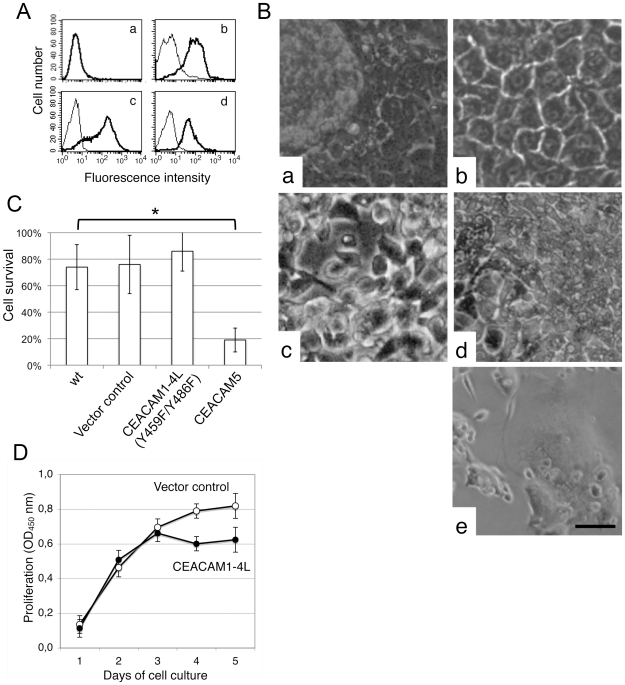
CEACAM1-L but not CEACAM1-S secures cell contact-inhibition. A) Analyses of the cell surface expression of different CEACAMs in the non-confluent log phase cultured parental A549 cells stably transfected with an empty vector (a), CEACAM1-4L (b), the mutant form of the intracellular ITIM motif CEACAM1-4L-Y459F/Y486F (c) and CEACAM5 (d). Samples were analyzed by flow cytometry using mAbs that specifically bind the different CEACAMs (thick line) or isotype matched control antibody (thin line) followed by FITC-conjugated secondary antibody. Data show one of three different, representative stably transfected A549 clones. B) Representative phase contrast images demonstrating the morphology of A549 cells stably transfected with empty vector (a), plasmids encoding for CEACAM1-4L (b), the ITIM mutant form CEACAM1-4L(Y459F/Y486F) (c), CEACAM5 (d) and CEACAM1-4S (e). Bar, 50 µm. C) Percentage of viable cells determined by the flow cytometry based annexin V-FITC/PI approach as described in Materials and Methods using cell spheroidals and aggregates harvested from the culture supernatant of wild type (wt) A549 cells and A549 cells transfected with control vector, CEACAM1-4L (Y459F/Y486F) or A549-CEACAM5. Data are shown as the percentage of viable cells as the mean of three independent experiments +/− Standard deviation. D) Growth properties of control vector transfected and CEACAM1-4L transfected A549 cells as determined by the MTS based method as described in Materials and Methods. A549- vector (white circles) and A549-CEACAM1 transfected cells (black circles) were seeded into in 96-well cell culture plates at a density of 25,000 cells/well. Following standard cell culture for 1, 2, 3, 4 and 5 days, respectively, the tetrazolium compound MTS was added and the samples were incubated at 37°C in a humidified, 5% CO2 atmosphere for 4 h. Experiments were performed in triplicate and results presented are expressed as means of OD 490 nm ± SD (*p≤0.005). The data show one representative result of three independent repeats of the experiment.

To analyze the functional impact of the different CEACAMs, we altered their expression patterns by stable transfection of parental A549 cells with CEACAM1-4L, CEACAM1-4S, the ITIM-mutant form of CEACAM1 [CEACAM1-4L(Y459F/Y486F)], CEACAM5 and CEACAM6, respectively. Since proliferating A549 cells did not express CEACAMs, the transfection success could be monitored by flow cytometry utilizing exponentially growing A549 transfectants. Empty vector transfected A549 cells served as a control and showed no expression of CEACAMs in the proliferative stage (Figure 5A, a). In contrast, A549-CEACAM1-4L (Figure 5A, b), A549-CEACAM1-4L(Y459F/Y486F) (Figure 5A, c) and A549-CEACAM5 (Figure 5A, d) cell lines revealed a strong exogenous CEACAM expression, respectively. The capability to produce A549-CEACAM5 cell lines demonstrated that in principle A549 cells are able to express GPI-anchored CEACAMs on their cell surface. Unexpectedly, we were not able to establish stable transfectants of A549-CEACAM1-4S and A549-CEACAM6, although the individual vectors transfected into HeLa cells resulted in CEACAM1-4S and CEACAM6 expression, respectively, proving the efficacy of these vectors (data not shown). Repeated transfections of A549 cells with CEACAM1-4S and CEACAM6 were carried out resulting in A549 cells, which were no longer able to exhibit normal proliferation. Analyzes by phase-contrast microscopy revealed that A549-CEACAM1-4S and A549-CEACAM6 cells, which initially survived the G418-selection procedure, detached from the culture vessel and subsequently died (data not shown). However, in contrast to A549-CEACAM6, several A549-CEACAM1-4S cells remained attached on the cell culture vessel and partially formed giant cells containing several nuclei (Figure 5B, e). This finding implied a malfunction of the regular cell division processes caused by over-expression of CEACAM1-4S. Compared with un-transfected and control-vector transfected A549 cells, A549-CEACAM1-4L exhibited a marked increase of the homogeneous, cobblestone-like epithelial cell morphology of a well spread monolayer, suggesting a potentiation of contact inhibition (Figure 5B, b). Importantly, none of the CEACAM1-4L over-expressing cell lines piled up from the confluent monolayer, and no spheroidal cell aggregates were formed. To determine, whether an intact cytoplasmic tail of CEACAM1-4L is essential for mediating the improved contact inhibition found in A549-CEACAM1-4L, un-separated A549 cells were stably transfected with an expression plasmid coding for a cytoplasmic ITIM mutant form of CEACAM1-4L[3], [30]. We found, that unlike CEACAM1-4L over-expressing cells, A549 cells carrying the mutant plasmid CEACAM1-4L (Y459F/Y486F) failed to grow as a homogenous monolayer. These cells barely established cell-matrix and cell-cell-contacts and appeared mainly as rounded cells. They grew as scattered, slightly attached cell-aggregates without forming solid monolayers (Figure 5B, c). Additionally, a number of A549-CEACAM1-4L (Y459F/Y486F) cells grew in suspension (data not shown), supporting an essential role of CEACAM1-4L in the mediation of contact inhibition of cell proliferation.

Finally, morphological analyses of the A549-CEACAM5 cells revealed that they grew as tight epithelial-like monolayers, although in contrast to the CEACAM1-4L over-expressing cells, A549-CEACAM5 cells tended to pile up on the monolayer, implying continued cell proliferation (Figure 5B, d). However, unlike un-transfected and control vector transfected cells, they did not form spheroidal cell aggregates, but displayed a number of single cells with a shriveled appearance on the top of the monolayer (Figure 5B, d). Subsequent flow cytometric apoptosis assays of the cells harvested from the different culture supernatants revealed that the survival of detached A549-CEACAM5 cells was significantly decreased compared with detached wild type A549, A549-control vector and A549-CEACAM1-4L(Y459F/Y486F) cells (Figure 5C).

### Different CEACAMs Are Involved in the Modulation of A549 Cell Proliferation

Next we investigated whether the expression of different CEACAMs promotes cell expansion, by measuring cell proliferation rates. We detected no significant alterations in the proliferation rates of parental A549 cells and A549 cells transfected with a control vector (Figure 5D, open circles), CEACAM1-4L (Figure 5D, filled circles), CEACAM1-4L(Y459F/Y486F) and CEACAM5 (data not shown) during the first two days after plating the cells at low cell number. Interestingly, after becoming confluent on day 3, A549-CEACAM1-4L cells stopped proliferating (Figure 5D, filled circles), whereas A549-control vector (Figure 5D, open circles), A549-CEACAM1-4L (Y459F/Y486F), and A549-CEACAM5 cells, continued to proliferate (data not shown). As an additional marker of cellular action we measured the acidification of the cell culture supernatant. Normally, the pH value of the cell culture supernatant of tightly confluent contact inhibited cells stays neutral, but decreases in non-contact inhibited cultures due to sustained metabolism. Therefore, we determined the pH values in the 5 days cell culture supernatants of the different confluent A549 cell lines. We detected a significant drop from pH 7.4 to about pH 6.6 in the supernatant of tightly confluent wild type A549, A549-control vector, A549-CEACAM1-4L (Y459F/Y486F) and A549-CEACAM5 cells, but not of confluent A549-CEACAM1-4L cells (data not shown). Thus, our results indicate that CEACAM1-4L negatively regulates proliferation of A549 cells by maintaining contact inhibition via its ITIM domain.

### CEACAM6 Induces A549 Cell Proliferation and Interferes with CEACAM1-4L Mediated Contact Inhibition

**Figure 6:**
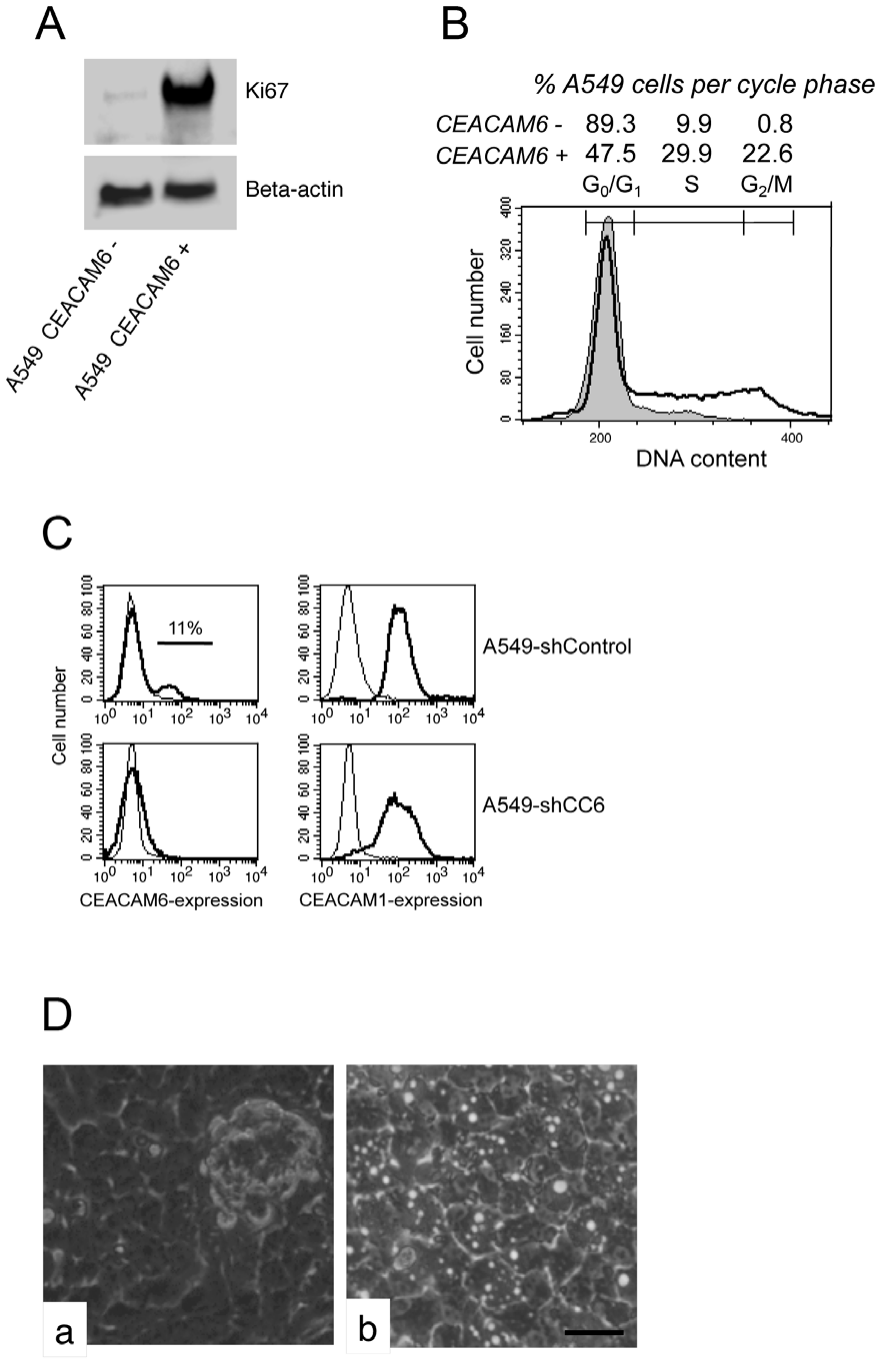
CEACAM6 acts as an inducer of cellular proliferation in confluent A549. A) The expression of the proliferation marker Ki67 is limited to A549-T cells that also expressed CEACAM6 on their cell surface. Confluent CEACAM6-negative and CEACAM6-positive A549-T cells were separated by mAb 9A6 loaded magnetic protein G microbeads and μMAS magnetic sorting columns (Miltenyi Biotec) as described in Materials and Methods. Immunoblot analysis of confluent CEACAM6-negative and CEACAM6-positive A549-T cell lysates was performed applying a Ki67 specific antibody followed by HRP-coupled secondary antibody and ECL detection. Beta-actin served as a loading control. The data shown are representative of three separate experiments. B) Cell cycle analysis of CEACAM6 negative and CEACAM6 positive A549-T cells. CEACAM6-negative and CEACAM6-positive A549 cells were fixed in 80% ethanol and stained with propidium iodide, and analyzed by flow cytometry as described in “Materials and Methods.” The DNA content in the different cell fractions is given in arbitrary units on the X-axis. Cells in the G2-M phase (second peak) contained twice as much DNA as cells in the G0-G1 phase (first peak). Cells between the peaks represent cells in the S-phase. The relative proportions of cells in the various phases are shown above the DNA profiles. Filled curve, CEACAM6 negative A549 cells; thick curve, CEACAM6 positive A549 cells. C) Confluent control sh-plasmid transfected A549-T (A549-shControl) and shCEACAM6 transfected A549-T cells (A549-shCC6) were stained for CEACAM1 with mAb clone 283340 and for CEACAM6 with mAb 9A6 (thick lines). The background fluorescence was determined by incubating the cells with control IgG antibody (thin lines). Samples were analyzed by flow cytometry. Compared to A549-shControl cells, A549-shCC6 cells completely lacked CEACAM6 expression, but continued to express CEACAM1. D) Phase contrast images of (a) control sh-plasmid transfected A549-T and (b) shCEACAM6 transfected A549-T cells. Confluent control sh-plasmid transfected A549-T piled up and formed unanchored spheroidal cell aggregates on top of the monolayer revealing insufficient contact inhibition. (b) In contrast, A549-shCC6 cells formed well spread monolayers without detection of unanchored, spheroidal cell growth indicative tight contact inhibition. Bar, 50 µm.

In confluent A549-NT cells only CEACAM1 was expressed on the cell surface. However, 10–15 per cent of the A549-T cells also expressed CEACAM6 (Figure 2A). Therefore, we assumed that the expression of CEACAM6 might be of importance for the induction of proliferation by overcoming the CEACAM1-4L mediated contact inhibition. To investigate this hypothesis, confluent A549-T cells were divided by magnetic bead separation into CEACAM6-negative and CEACAM6-positive subpopulations. Subsequently, the expression levels of the proliferation marker Ki-67 were evaluated by immunoblotting. As shown in Figure 6A, only the CEACAM6-positive subpopulations of confluent A549-T revealed a significant level of Ki-67 suggesting involvement of CEACAM6 in the induction of cell proliferation. To further investigate this observation, we determined the growth phase of the cells by measuring the DNA content as an additional marker of proliferation. Our results confirmed that CEACAM6 negative A549 cells were found only in the G0/G1 phase, whereas CEACAM6 positive A549 cells showed active cell cycling with high proportions of cells being in both the S-phase and the G2/M phase (Figure 6B). To further substantiate our results, we performed RNA interference experiments by stable transfection of A549-T cells either with scrambled sh-control plasmids or with shCEACAM6-plasmids. Flow cytometric (Figure 6C) and ELISA analyses (data not shown) of A549-T cells transfected with the different shCEACAM6-plasmids confirmed the complete absence of CEACAM6 expression in the confluent growth stage, while CEACAM1 was still expressed. Next, we investigated the morphology of the monolayer in the confluent stage and found, that A549-T-sh CEACAM6 monolayers had a cobblestone-like morphology consisting of closely associated cells, comparable to the morphology found in the A549-NT and A549-CEACAM1-4L cells (Figure 6D). Moreover, in confluent CEACAM6 shRNA plasmid transfected A549 cells neither spheroidal cell clusters nor media acidification, indicating impairment of contact inhibition, was observed.

## Discussion

In the present study, we provide new insights into the multiplicity and diversity of CEACAM1, CEACAM5 and CEACAM6 expression and their functions in tumor development and progression. We found, that CEACAM1 but not CEACAM5, 6, or 7 was expressed on the surface of A549 human alveolar lung epithelial cells. However, this CEACAM1 expression was limited to the post-confluent contact inhibited growth stage, whereas proliferating cells did not show any CEACAM expression on the cell surface. In addition to the expression of CEACAM1 in contact-inhibited A549 cells, significant amounts of soluble variants of CEACAM5 and CEACAM6 were released by confluent A549 cells, thus exhibiting a likely source for increased CEACAM5 and CEACAM6 serum levels, which are frequently detected in cancer patients[31]–[33].

Further important insights from this study rely on the characterization of two functionally distinct A549 subpopulations, named A549-NT and A549-T. Cells of the A549-NT subpopulation retains the characteristics of a more differentiated, contact-inhibited cell type without tumorigenic effects in the nude mouse system, whereas A549-T cells exhibit a relatively undifferentiated, anchorage-independent cell growth. Accordingly, cells of this subpopulation appear to be highly tumorigenic in nude mice[3]. Loss of contact inhibition and the gain of anchorage-independent cell growth are hallmarks of undifferentiated cancer cells in vitro^6^. Thus, the A549 cell line is an excellent model system to investigate normal and malignant cellular processes and tissue architecture, which are known to be largely established and maintained by members of the CEACAM family. In our study analyses of the two A549 subpopulations reveals that CEACAM1-4L negatively regulates proliferation by maintaining contact inhibition via its ITIM-domain. These findings are in accordance with previous reports describing CEACAM1 as a tumor-suppressor gene[34]. Interestingly, a fraction of tightly confluent A549-T cells also expressed CEACAM6 on the cell surface. These CEACAM6 positive A549-T cells also stained positive for Ki-67 and showed a DNA-based cell cycle profile characteristic for dividing cells, two well-known markers of cellular proliferation. Our data indicate that CEACAM6 expression on the surface of A549 cells abolishes the cell-cell contact-triggered inhibitory signal mediated by CEACAM1. Consequently, we conclude that CEACAM6 expression on the cell surface acts as a potent inducer of cellular proliferation. Moreover, these observations are of central importance for explaining the differences in growth behavior and tumorigenicity of A549-NT and A549-T cells. These findings demonstrate that the interplay between different CEACAMs is crucial for the regulation of cell proliferation and growth arrest.

Furthermore, over-expression of CEACAM1-4L strengthened contact inhibition and the formation of tightly confluent monolayers which were dependent on the presence of an intact ITIM domain in the cytoplasmic part of CEACAM1-4L. Our data are in accordance with several reports indicating tumor suppressive functions when the CEACAM1-4L gene was over-expressed in prostate, bladder, colon and breast cancer cells. In these studies, over-expression of CEACAM1-4L lead to significantly lower growth rates and less tumorigenicity in both in vitro and in vivo models when compared with un-transfected tumor cells [32]–[36]. In addition, we found that A549 cells over-expressing the cytoplasmic S-isoform of CEACAM1 on their cell surface completely lost the ability to grow as an epithelial monolayer. These data correspond to a report of Wang et al. [36]. In this study the authors analyzed tumor tissue and corresponding normal lung tissue from patients with non-small cell lung cancer (NSCLC) and found reduced expression levels of the L-form of CEACAM1 as well as increased levels of the S-form of CEACAM1 in most of the lung cancer tissues^36^. Changes in the isoform ratios of CEACAM1 favoring the S form has already been demonstrated to increase proliferation in cancer cells [37], [38]. However, in our investigation we did not find a significant difference in the L/S ratio expressed by the two A549 subpopulations that would account for the differences in the observed growth pattern.

As mentioned above, in contrast to the A549-NT cells, in addition to CEACAM1, about 15 percent of the A549-T cells also expressed CEACAM6 on their cell surface, and the alterations in contact inhibited growth pattern could be reversed in A549-T cells by shRNA mediated knock down of CEACAM6 expression. CEACAM6 expression has already been inversely correlated with cellular differentiation and has been identified as a marker for decreased survival in patients with CEACAM6 expressing cancers.[35], [37], [38] In a series of experiments, Duxbury and associates have demonstrated that CEACAM6 over-expression in pancreatic cancer is a determinant of cell proliferation and cell invasiveness[39]. On the other hand, targeting CEACAM6 results in decreased tumor growth, and inhibits metastases in a mouse xenograft model[40]. Our results are in accordance with these findings, but in addition we show for the first time that CEACAM6 acts as an inducer of proliferation in A549 cells, putatively by interfering directly or indirectly with the contact-inhibiting signal triggered by CEACAM1-4L. These data also support previous data implying that CEACAM6 over-expression inhibits the tissue architecture surveillance mechanism known as anoikis[41]. In principle A549 cells were able to express GPI anchored CEACAMs as demonstrated by the generation of A549-CEACAM5 cell lines (Figure 5A). However, our finding that the survival of detached A549-CEACAM5 cells was significantly decreased was in contrast to the results published by Camacho-Leal and Stanners, who demonstrated that the GPI anchor of CEACAM5 mediates anoikis inhibition[42]. This different result may be based on the fact that A549 cells represent a human cell system endogenously expressing CEACAMs whereas Stanners group utilized L6 rat myoblasts, which normally do not express CEACAM5. In addition, in rat no GPI-linked CEACAMs were described so far.

Our observations seem to be in contrast to the report of Laack et al., who identified CEACAM1 expression as a prognostic marker for poor outcome in patients with adenocarcinomas of the lung[43]. However, Sienel et al. suggested that the unfavorable prognostic influence of CEACAM1 might be derived from its angiogenic influence leading to an increased angiogenic activity and micro vessel density (MVD) in non-small-cell lung cancer. It is tempting to speculate that the expression of other CEACAMs in NSCLC cells could overcome the CEACAM1-mediated contact inhibition in these cells.

Taken together, several members of the CEACAM family play important roles in tumorigenesis and the development of metastatic disease[10], [14]. In addition, each CEACAM and each CEACAM splice variant can exhibit distinct functions, and interactions between different CEACAMs seem to play a central role in the fine-tuning of several cellular functions[12], [38]. Nevertheless, in most studies of the role of CEACAM expression in cancer cells, the expression and function of only one CEACAM was investigated[11], [12], [22], [34]–[36], [40], [44]–[52]. The results of our study show that changes in the expression patterns of different CEACAMs can alter their diverse functions on cell-cell interactions leading to changes in differentiation and survival, as well as tumorigenicity. Thus, future studies should focus on the expression patterns and the functional interplay of all CEACAMs and CEACAM isoforms expressed on the investigated tissues.

## Materials and Methods

### Antibodies and Reagents

The media and media supplements were purchased from Gibco-Life Technology (Eggenstein, Germany) and the chemicals were obtained from Sigma (Taufkirchen, Germany), unless stated differently.

The hybridoma secreting the mouse mAb 18/20 (specific for human CEACAM1/3/5) was recently described[20]. The mAb Col-1 was purchased from Invitrogen (Carlsbad, CA) and the anti-human CEACAM1 mAb clone 283340 from R&D systems (Minneapolis, MN). Anti-human CEA/CEACAM5 mAb clone CI-P83-1 was obtained from Dianova (Hamburg, Germany). The mAbs 9A6 (specific for CEACAM6) and BAC2 (specific for CEACAM7) were provided by Genovac (Freiburg, Germany). The CEACAM8-specific mAb 80H3 was obtained from Coulter International (Miami, FL). The rabbit anti-CEA (binding CEACAM1/3/4/5/6/7/8) and the polyclonal antibody for Ki-67 were obtained from Dako (Copenhagen, Danmark). The anti-beta-actin-peroxidase antibody (clone AC-15) was purchased from Sigma. FITC- and HRP-coupled secondary goat anti-mouse IgG F(ab)_2_ fragments were obtained from Jackson ImmunoResearch (West Grove, PA).

### Cells

A549 epithelial cells (type II alveolar lung epithelium cells; ATCC CCL85) were cultured in DMEM (GIBCO) supplemented with 10% (v/v) heat inactivated fetal calf serum (FCS) and 4 mM L-glutamine at 37°C in a humidified atmosphere with 5% CO_2_. A549 cells analyzed as growing in log phase were in the mid-logarithmic phase of growth, while confluent cells were cultivated until tight cell-cell contacts were developed. The cell spheroids were collected from the supernatant of tight confluent growing cells. In all cases, cell viability was over 90% as measured by trypan blue exclusion. Changes in the cellular morphology during different growth stages were monitored by standard phase contrast microscopy utilizing the Leica DMIL-system (Leica Microsystems, Wetzlar, Germany) and the ProgRes Capture Pro2.5 analyses software (Jenoptik, Jena, Germany).

### Cell Separation

The separation of A549 cells into subpopulations was performed as described by Croce et al. Briefly parental exponentially growing cells were harvested by the standard trypsinization procedure and separated by Percoll density gradients (GE Healthcare). Thus, cells were re-suspended in 1.5 ml PBS and carefully layered on top of the Percoll gradient consisting of 1.5 ml of each stock solution of density 1.035, 1.040, 1.050, 1.060, 1.065, 1.070 and 1.080 g/ml prepared according to the manufacturer's protocol. The gradients were centrifuged at room temperature at 800 g for 40 min in a swinging-bucker rotor without using the brake. Fractions of 0.5 ml were collected from the top, and the samples were washed twice with DMEM. The cell number in each fraction was determined by counting in a Neubauer chamber and cell viability was determined by trypan blue exclusion. Cells were then plated and cultured as described above until further use.

For some experiments, CEACAM6-positive and CEACAM6-negative parental A549 cells were separated using magnetic protein G microbeads (Miltenyi Biotec, Bergisch Gladbach, Germany) coupled with the CEACAM6-specific mAb 9A6. Thus, cells were incubated with protein G-mAb 9A6 microbeads for 1 h in medium on ice. Next, cells were applied to μMAS magnetic sorting columns (Miltenyi Biotec) and the further separation steps were performed according to the manufacturer's protocol. The quality of separation was monitored by flow cytometry.

### Cell Proliferation Assay

For colorimetric determination of cell proliferation, the A549 cells and A549-CEACAM1-4L transfectants were plated in a 96-well cell culture plate at a density of 25,000 cells/well in triplicate. Following standard cell culture the tetrazolium compound MTS of the CellTiter96 Aqueous One assay (Promega Corp., Madison, WI) was added to each well. Then cells were incubated at 37°C in a humidified 5% CO_2_ atmosphere for 4 h. The absorbance of soluble formazan produced by cellular reduction of the MTS was measured at 490 nm using a microplate reader (Tecan, Craislheim, Germany). The quantity of formazan product, as measured by the amount of 490 nm absorbance, is directly proportional to the number of living cells in culture. Experiments were performed in triplicate and the results presented were expressed as the OD at 490 nm.

### Measurement of the Acidification in the Cell Supernatant

Cell culture acidification was determined utilizing cell-free supernatant fluid for estimating the pH value by the color change of the tracer in the media and by pH-indicator strips pH 5.2 – 7.2 (Merck, Darmstadt, Germany). Media was considered as acidified if the pH decreased from pH 7.3 to below pH 6.5.

### Transfection

A549 cell lines, permanently expressing CEACAM1-4L, CEACAM1-4S, CEACAM5 and CEACAM6, respectively, were generated. The pRC-CMV-based NEO-resistant expression plasmids encoding either CEACAM1-4L, CEACAM1-4S or a variant of CEACAM1-4L with mutations in the cytoplasmic ITIM (exchange of Y459 to F459 and Y486 to F486), (kindly provided by Prof. W. Zimmermann, University Clinic – Grosshadern, Ludwig-Maximilians-University Munich, Germany), CEACAM5-pRc/CMV (kindly provided by Dr. Grunert, Institute of Immunology, Freiburg, Germany) or CEACAM6-pdKCR-neo (kindly provided by Dr. M. Kuroki, Department of Biochemistry, Fukuoka University, Fukuoka, Japan) were transfected into parental A549 using lipofectamine 2000 according to the manufacturer's protocol (Invitrogen). Stable transfected cells were selected in culture medium containing 1 mg/ml of Geniticinsulfat (G418, Biochrom, Berlin, Germany). The surface expression of CEACAMs in individual clones growing in log phase was determined by flow cytometry. Three independent clones of each transfection were analyzed to confirm consistent behavior and receptor expression.

### Small/Short Hairpin RNA (shRNA) Interference in A549 Cells

Gene silencing of CEACAM6 was performed with pre-designed SureSilencing™ CEACAM6-shRNA plasmids (Super Array-Bioscience Corporation) containing four pooled shRNA sequences to ensure an effective depletion of CEACAM6 in A549 cells. The shRNA-CEACAM6 sequences were as follows:

Clone ID 1: 5′-ACGATGCAGGATCCTATGAAT-3′;

Clone ID 2: 5′-AACGATGCAGGATCCTATGAA-3′;

Clone ID 3: 5′-GAACGATGCAGGATCCTATGA-3′;

Clone ID 4: 5′-GTAGAGTGGTGCTGCTTTAAT-3′;

The scrambled 5′-GGAATCTCATTCGATGCATAC-3′ sequence (sh-CON) served as a control. The stable A549 shRNA transfection was carried out with Lipofectamine 2000 (Invitrogen) followed by selection for 2–3 weeks in 1 mg/mL G418-containing medium. Two control clones (sh-CON-A549) and three clones of CEACAM-silenced A549 cells were chosen and further characterized by flow cytometry and Western blot.

### Immunoblot Analysis

Cells were lysed in RIPA based lysis buffer [50 mM Tris-HCl, pH 7.5, 150 mM NaCl, 1% Triton X-100, 0.5% sodium deoxycholate) supplemented with protease inhibitor cocktail set III (Calbiochem) and PhosSTOP phosphatase inhibitor cocktail (Roche) on ice for 30 min. Lysates were centrifuged at 10,000 *g* at 4°C for 15 min and approximately 50 µg of total protein was subjected to SDS-PAGE, blotted to membrane (Schleicher & Schuell, Dassel, Germany) and reacted with anti-CEACAM1, anti-CEACAM5, anti-CEACAM6 and anti Ki67. Anti-beta-actin antibody was used as Western blot loading control.

### Relative and Quantitative Flow Cytometry

A549 cells (5×10^5^) were stained with mAbs (20 µg/ml) anti-CEACAM1 (clone 283340), anti-CEACAM5 (Col-1) anti-CEACAM6 (9A6), anti-CEACAM7 (BAC2) and anti-CEACAM8 (80H3) diluted in 3% FCS/PBS for 1 h on ice, washed with ice-cold PBS, and incubated with FITC conjugated anti-mouse F(ab')_2_. Background fluorescence was determined using isotype-matched Ig. For DNA quantification, the cells were fixed in ice cold 80% ethanol and then were incubated for 15 min at 37°C in PBS containing 100 µg/ml DNase-free RNase (Sigma), and 10 µg/ml propidium iodide. The stained cell samples were examined in a FACScalibur flow cytometer (BD Biosciences, San Diego, CA) and the data were analyzed utilizing the CellQuest software. Where applicable, dead cells, identified by PI staining, were excluded from the determination.

The absolute quantitative expression pattern of the different CEACAM-proteins was determined using QuiFiKit® (Dako, Glostrup, Denmark) according to the manufacturer's protocol. Hence, the calibration-bead suspension coated with known quantities of mouse IgG was incubated in parallel with the samples undergoing secondary staining procedure with FITC conjugated anti-mouse F(ab')_2_. Utilizing the median values of the calibration beads, a calibration curve was constructed and the absolute numbers of CEACAM-molecules per cell were calculated.

### Sandwich-ELISA

A549 cell culture supernatants were collected from cells grown to confluence plus 6 days (A549confluent). After centrifugation at 10,000×g, the samples were stored at −80°C until further use. The evaluation of CEACAM1, CEACAM5 and CEACAM6 was performed by sandwich-ELISA. In brief, 96-well flat bottom immunoassay MaxiSorpELISA plates (Nunc Maxisorp, Invitrogen) were coated overnight at 4°C with 1 µg/well rabbit anti-human CEA antibody (Dako). After three washes with PBS, plates were blocked with 350 µl/well of PBS/3% BSA for 1 h at room temperature. Thereafter, 100 µl/well of two-fold dilutions of supernatants in PBS/1% BSA were added. As positive antigen controls, cell lysates of Hela, Hela-CEACAM1-4L, Hela-CEACAM5, Hela-CEACAM6, Hela-CEACAM7 and Hela-CEACAM8 cells were added. Plates were then incubated at 4°C overnight. After washing, 1 µg/100 µl/well of mAb anti-CEACAM1 (clone 283340), anti-CEACAM5 (Col-1, CI-P83-1), anti-CEACAM6 (9A6), anti-CEACAM7 (BAC2) or anti-CEACAM8 (80H3) diluted in 1% FCS/PBS was added and incubated for 4 h at room temperature. After washing, HRP-conjugated anti-mouse F(ab')2 was added and incubated for 2 h at room temperature. Finally, the plates were washed 4 times, developed using TMB (Sigma) according to the manufacturer's protocol, and the absorbance was detected at 450 nm in a Sunrise-ELISA reader (Tecan, Crailsheim, Germany). All measurements were determined in triplicates.

### Quantitative RT- PCR

Specific primers (table 2) were designed to estimate the amount of four different CEACAM1 isoforms: CEACAM1-4L was detected with the CEACAM1-4 sense primer and CEACAM1-L antisense primer, CEACAM1-4S cDNA was amplified with CEACAM1-4 sense primer and CEACAM1-S antisense primer. The CEACAM1-3L cDNA was detected with the CEACAM1-3 sense primer and CEACAM1-L antisense primer and CEACAM1-3S cDNA was ascertained with CEACAM1-3 sense primer and CEACAM1-S antisense primer. The primers were purchased from Eurofins MWG Operon (Ebersberg, Germany). An internal standard for each CEACAM1 isoform was generated utilizing an endpoint PCR using 1 µl cDNA, 0.2 U Taq polymerase (Genecraft, Luedinghausen, Germany), 2 µmol/l dNTP, 3 µl 10x buffer, 0.5 µmol/l of the respective sense and antisense primers (table 2) in a final volume of 30 µl under standard conditions (95°C for 3 min followed by 34 cycles at 95°C for 30 sec, 60°C for 45 sec and 72°C for 30 sec and an extension at 72°C for 8 min). Afterwards the PCR products were purified with PCR purification kit (Qiagen, Hilden, Germany) and cloned in the pDrive cloning vector with the PCR cloning kit (Qiagen, Hilden, Germany) according to the manufacturers protocol. Colonies were picked and the presence of the PCR product in the isolated DNA was verified by sequencing. The appropriate plasmid containing the PCR product was used as a specific qPCR standard.

**Table 2:** Primer sequences used in RT-PCR.

Gene	Primer name	Sequence
CEACAM1	CEACAM1-4	ACC CTG TCA AGA GGG AGG AT
	CEACAM1-L	TGA GGG TTT GTG CTC TGT GA
	CEACAM1-3	TAG TCA CTG ATA ATG CTC TAC C
	CEACAM1-S	GTC CTG AGC TGC CGG TCT

The total RNA of A549 cells was isolated using the Qiagen RNeasy Kit. Afterwards 1 µg of each sample was reverse transcribed with the QuantiTect Reverse Transcription Kit (Qiagen) in a final volume of 20 µl. For each gene a qPCR was performed by using 1 µl of cDNA, the adequate standard-DNA (10^3^–10^8^ molecules) and blanks with the SYBRgreen master mix (Bio-Rad) and suitable primers (see above) in a final volume of 25 µl according to the manufacturers protocol with the IQ5 cycler (Bio-Rad). The qPCR products were visualized using 3% agarose gel/Tris-acetate-EDTA separation and ethidium bromide staining, and then the total amounts of molecules/well were determined using the IQ5 software (Bio-Rad) the GenEx professional software (Multid, Göteborg, Sweden).

### Statistical Analysis

Where applicable, data are presented as the mean ± SD. Statistical significance was determined using the Student's t-test. Differences were considered to be statistically significant at a *P* value of <0.05.
